# A novel theory of ageing independent of damage accumulation

**DOI:** 10.18632/aging.204956

**Published:** 2023-07-28

**Authors:** James Wordsworth, Daryl Shanley

**Affiliations:** 1 Newcastle University Biosciences Institute, Newcastle University, Newcastle upon Tyne, United Kingdom

**Keywords:** ageing, evolution, damage, cell competition, metabolic slowdown

The underlying cause or causes of ageing are an enduring mystery, but in 1977 Kirkwood postulated that organisms might gain a fitness advantage by reducing investment in somatic maintenance if this allowed them to invest more resources in more crucial processes such as reproduction [1]. The accumulation of somatic damage was therefore inevitable, and his disposable soma theory has dominated gerontology ever since. However, as our understanding of ageing increases, it is becoming increasingly difficult to align all the aspects of ageing with accumulating damage. For example, mutations that increase damage accumulation can also increase longevity [2], while rejuvenation revelations such as parabiosis and Yamanaka factors indicate that youthfulness can be regained without high energetic cost and despite high levels of damage [3].

We recently published selective destruction theory (SDT), which suggests a mechanism of ageing which is both independent of accumulating damage and consistent with epigenetic rejuvenation [4]. We argue that in multicellular organisms, neighbouring cells are in constant competition. When mutations arise that increase a cell’s growth rate, they bestow a selective advantage (an extreme example would be cancer, but most will not be). If these cells are uncontrolled, their growth advantage will allow them to spread, and their overactive metabolism could result in a host of detrimental or even lethal overactivity disorders. For example, in β-cells where growth is tied to insulin production, fast mutants spreading could produce a lethal drop in blood glucose. Another less tissue-specific example is the increased propensity of fast growing/metabolising fibroblasts to reach the critical threshold required for fibrosis [5]. Lastly, fast mutants are also likely to be more tumorigenic, while slow mutants will be less active, less fibrotic, and less tumorigenic even compared to wildtype cells. We therefore proposed that a maintenance mechanism which selectively destroyed fast cells might undergo positive selection even if it caused the spread of slow mutants as it would reduce the risks of overactivity disorders.

**Figure 1:**
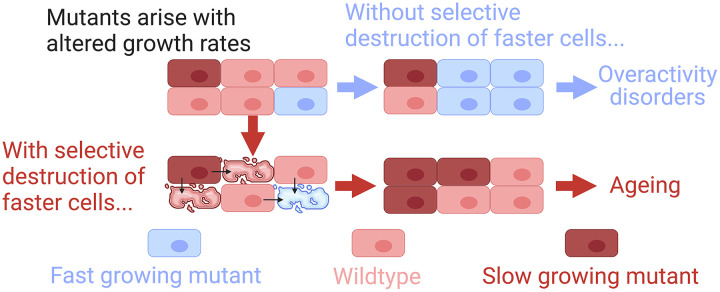
**Outcomes of cell competition and control by selective destruction.** Created with biorender.com.

We used computational agent-based modelling of a hypothetical tissue in NetLogo to compare the outcomes of selective destruction (preferentially removing fast cells) compared to unselective destruction which attempted to remove fast mutants and slow mutants with equal proficiency (Figure 1). Importantly, the only difference between these two mechanisms was that selective destruction was less capable of removing slower cells, while both mechanisms were equally effective at removing faster cells. Our results indicated that only selective destruction could prevent the eventual dominance of faster cells.

This reflected that selective destruction provided an important force of counterselection, balancing the growth advantage of faster cells with a survival advantage for slower cells. Strengthening selective destruction reduced the risk of overactivity disorders, but increased the spread of slower cells (Figure 1). We predicted that this would result in a gradual metabolic slowdown, which could reflect the underlying cause of ageing.

For ageing (inducing genes) to undergo positive selection, the benefits of reduced overactivity disorders would need to outweigh the costs of age-related decline; as the effects of ageing would occur later, we predicted selective destruction would be antagonistically pleiotropic.

Metabolic slowdown is one of the key phenotypes of ageing. Cells divide more slowly, mitochondria become less efficient at producing ATP, and our organs atrophy and turn to fat. The dominant explanation for this is the accumulation of damage obstructing metabolism. Indeed, critics of SDT will observe that the occurrence of fast and slow mutants requires molecular damage. Likely, this is true, although epigenetic differences in growth could potentially arise from positional differences relative to nutrient supplies, or spatial inconsistency in growth signals. If damage is involved, single point mutations are sufficient to radically affect growth, and the rate they are produced would need to be significantly reduced to affect ageing. We would argue that the absence of such fidelity need not reflect the energetic costs of maintenance, which would be minor compared to the costs associated with the necessary reduction in cell division rate. Once these faster mutants are present in the tissue, their spread does not reflect additional damage (accumulation), but positive selection. Indeed, the maintenance mechanism required to stop the spread of fast mutants is selective destruction, increasing the strength of which will only accelerate ageing.

The mechanism of selective destruction is currently theoretical. In our most developed model, we demonstrated that if slow cells induced epigenetic changes in faster cells causing their metabolism to slow (rather than killing them) it not only reduced unnecessary cell death, but also further reduced the likelihood of overactivity disorders by preventing the spread of fast cells. The resulting epigenetic growth suppression could therefore reflect a kind of ageing program designed to prevent overactivity disorders, and may explain why the methylation of specific CpG islands provides such accurate ageing clocks. It would also explain epigenetic rejuvenation by Yamanaka factors and parabiosis, so we predict that methylation of CpG islands will affect cell growth.

Arguably, in post-mitotic organisms suppressing cell growth is not necessary and therefore neither is selective destruction. However, given the myriad advantages associated with cell proliferation (eg. replacing damaged cells) it is worth asking why these organisms have evolved to become post-mitotic. We would speculate that the primary reason is to escape the disadvantages of cell division, i.e. overactivity disorders. As such, the post-mitotic state would reflect the selective destruction of every proliferating cell.

A wider definition of selective destruction could therefore include any process designed to slow, stop, or remove fast metabolising cells and reduce overactivity disorders. One known mechanism is the deactivation of telomerase, which has already been suggested to be an anti-cancer mechanism by limiting the division of highly replicative cells through the depletion of telomeric DNA [6]. Although cancer is just one of the overactivity disorders made more likely by the spread of faster cells, the idea that ageing reflects a trade-off with cancer is longstanding [7]. SDT suggests an additional mechanism by which the suppression of cancer cells may induce ageing through metabolic slowdown. However, it should be noted that whole-body ageing need not be so clear cut as universal slowdown. Faster cells escaping control will accelerate metabolism in some tissues, and different organismal life histories may adopt different points on the balance between overactivity and slowdown, maximising fitness within their specific niche. For example, smaller organisms requiring fewer cell divisions could expect fewer fast mutant cells to arise and therefore reduce selective destruction. These organisms would not necessarily be more cancer prone, but the cancers that arose would occur in much faster metabolising cells, making them more aggressive, which may underly some of the differences between tumour cells of mice and humans [8].

In conclusion, selective destruction could reflect a proximal cause of ageing in the form of metabolic slowdown. It is not dependent on damage accumulation or the costs of maintenance, although it is not exclusive with these theories. However, anti-ageing therapies directed against selective destruction would have the significant problem that they would increase the risk of overactivity disorders such as cancer.
